# Marantic Endocarditis in Malignancy: A Case Report of a Challenging Diagnosis

**DOI:** 10.7759/cureus.91174

**Published:** 2025-08-28

**Authors:** Inês Ferreira, Inês Fiúza M. Rua, Diogo Ramos, Sérgio Cabaço, André Valente

**Affiliations:** 1 Internal Medicine, Unidade Local de Saúde de São José, Lisbon, PRT

**Keywords:** hypercoagulability states, infectious endocarditis, lung cancer, marantic endocarditis, stage four lung cancer, vegetations

## Abstract

Marantic endocarditis is a condition characterized by sterile vegetations in the heart valves, and is much rarer than infectious endocarditis. It’s typically associated with conditions such as cancer or autoimmune diseases. We report the case of a patient with stage four lung cancer, presenting with fever after chemotherapy, and diagnosed with marantic endocarditis, after extensive testing to exclude an infectious agent. The patient’s imaging techniques suggested infectious embolization to the liver and spleen, which supported the diagnostic hypothesis of infectious endocarditis, but the liver biopsy was positive for metastasis. Oncology was consulted for follow-up, and the patient was discharged on anticoagulation therapy, the standard treatment for marantic endocarditis. This case underscores the difficulty of diagnosing marantic endocarditis and the extensive testing required, considering its poor prognosis and the importance of treatment.

## Introduction

Marantic endocarditis (ME) is a rare condition characterized by sterile vegetations in the heart valves, and it is usually associated with hypercoagulability states, such as cancer or autoimmune diseases [1]. Cancer is a leading cause of ME [2], and it has been shown in several studies that malignant neoplasms are found in a high percentage of patients with ME (59% in one cohort) [3]. Lung cancer, specifically, has been shown to be the leading neoplastic cause of ME in another study [4], with the probability increasing when it is a metastatic cancer.

Unlike infective endocarditis, where valve vegetations contain bacteria, ME forms sterile vegetations of fibrin and platelets. Clinically, ME often differs from infective endocarditis because patients may lack fever and other classic signs of infection. While ME is often associated with systemic embolic events, such as ischemic strokes, renal or splenic infarctions, cases without embolic manifestations are exceedingly rare. The absence of embolic events in ME presents a diagnostic challenge, as it can mimic other conditions, leading to delays in appropriate management.

Neurological emboli, particularly ischemic strokes, are among the most common and devastating complications, frequently associated with high morbidity and mortality [5]. Recurrent embolization, even under appropriate therapy, further contributes to poor outcomes. Importantly, because ME often arises in the context of advanced malignancy, embolic events can accelerate clinical deterioration and shorten survival, compounding the already limited prognosis associated with the underlying disease.

## Case presentation

A 53-year-old male presented to the emergency department (ED) with fever (measured axillary temperatures above 38ºC) and aqueous nasal discharge. The patient had a history of recently diagnosed lung cancer, with a single cerebral metastasis that had been removed a month prior to admission. At the time of presentation, he was undergoing his first cycle of chemotherapy, having had an administration five days prior to admission.

On initial examination in the ED, the patient was alert, oriented, and hemodynamically stable. On auscultation, breath sounds were clear and equal bilaterally, and no heart murmur was noted. Neurological examination revealed no deficit despite the recent brain surgery. Blood work (Table 1) was significant for an increased leukocyte count with neutrophilia and elevated C-reactive protein (CRP). Blood cultures were taken, and the patient was admitted to inpatient care for intravenous (IV) large-spectrum antibiotics and investigation, considering a likely post-chemotherapy infection.

**Table 1:** Laboratory parameters at emergency department admission

Laboratory parameter	Patient values	Reference range
Leucocytes	14.97 x 10^9^/L	4.5 - 11.0 x 10^9^/L
Neutrophils	10.69 x 10^9^/L	2.0 - 8.5 x 10^9^/L
C-reactive protein (CRP)	240.9 mg/L	< 5.0 mg/L
Blood cultures (5-day incubation)	Negative	_
Urine culture (flow cytometry)	Negative	_

During hospitalization, blood work (Table 2) for infectious diseases ruled out cytomegalovirus, Epstein-Barr virus, hepatotropic viruses (hepatitis C virus and hepatitis B virus), human immunodeficiency virus, syphilis, and parvovirus B19. Repeated blood and urine cultures were negative. A serum procalcitonin level of 0.32 ng/mL was obtained; while not markedly elevated, it was insufficient to conclusively rule in or rule out a bacterial infection. Considering the initial complaints of aqueous nasal discharge, a respiratory virus panel was also taken, and came back negative.

**Table 2:** Inpatient investigation for infectious agents NAAT: nucleic acid amplification test; PCR: polymerase chain reaction; BAL: bronchoalveolar lavage; LJ: Lowenstein-Jensen; CMV: cytomegalovirus; EBV: Epstein-Barr virus; VCA: viral capsid antigen; EBNA: Epstein-Barr virus nuclear antigen; EA: early antigen; HBs: hepatitis B surface antigen; HCV: hepatitis C virus; VDRL: Venereal Disease Research Laboratory;

Laboratory parameter	Patient result
Blood cultures (5 sets on different days)	Negative
Urine cultures (3 samples on different days)	Negative
Bacterial DNA in the blood (PCR)	Negative
Fungal DNA in the blood (PCR)	Negative
BAL culture	Negative
NAAT for* Mycobacterium tuberculosis* in BAL	Negative
PCR for *Pneumocystis jirovecii* in BAL	Negative
Blood culture in LJ medium	Negative
BAL culture in LJ medium	Negative
Anti-CMV IgG and IgM	Negative
EBV-VCA IgG and IgM	IgG positive, IgM negative
EBNA-IgG	Positive
EBV-EA-IgG	Negative
Huddleson test	Negative
Brucella Rose Bengal test	Negative
Anti-Parvovirus B19 IgG and IgM	Negative
HBsAg	Negative
Anti-HCV total antibodies	Negative
Anti-HIV 1+2 antibodies (fourth generation)	Negative
VDRL test	Negative
Anti-*Echinococcus granulosus *antibodies	Negative
Anti-*Coxiella burnetii* IgG and IgM	Negative
Anti-*Mycoplasma pneumoniae* IgG and IgM	Negative

**Figure 1:**
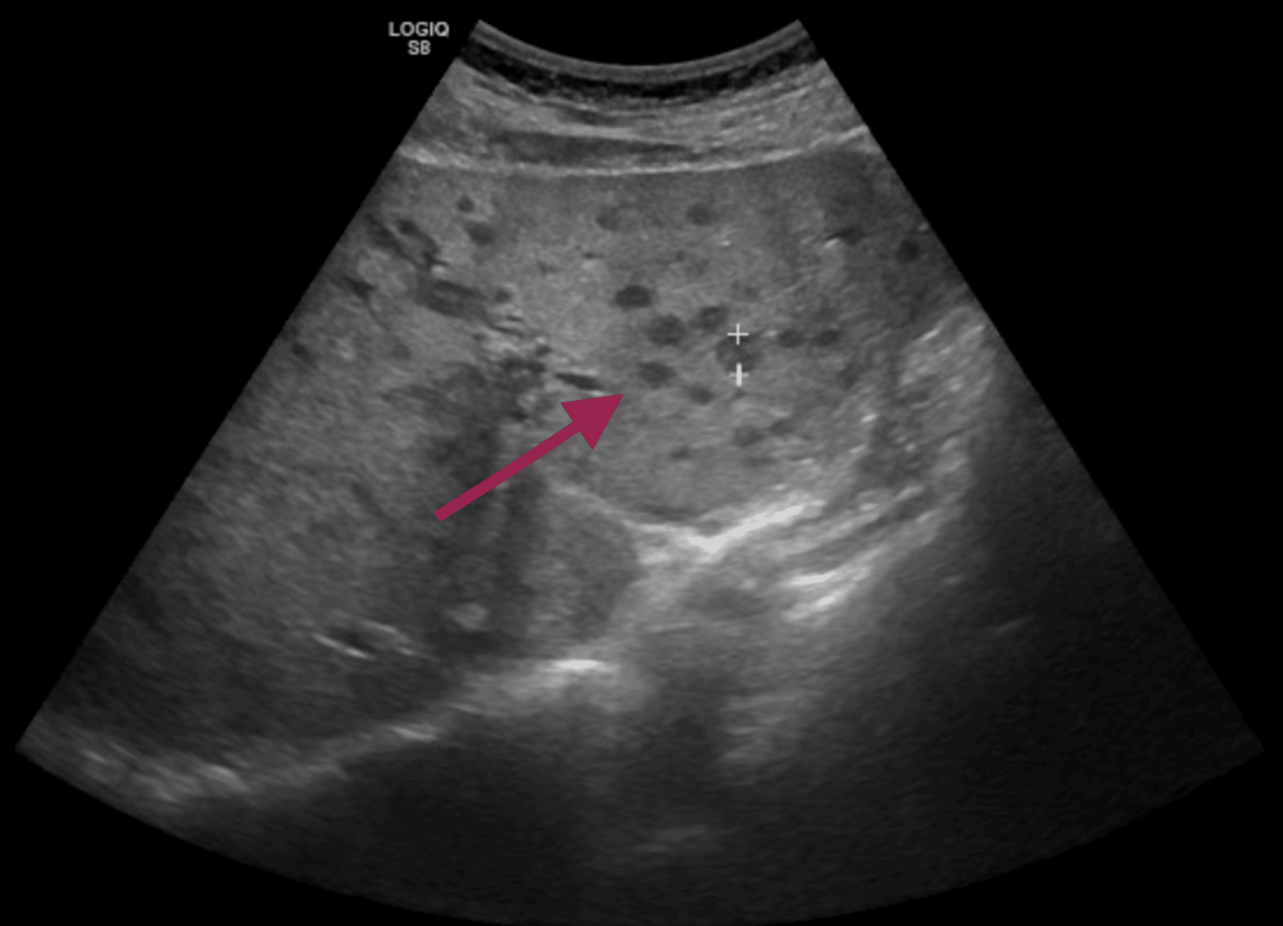
Abdominal ultrasound showing multiple hepatic nodules suggestive of infeccious foci Abdominal ultrasound showing hepatic nodules that were first thought to be infection but later found to be metastases, after biopsy.

**Figure 2:**
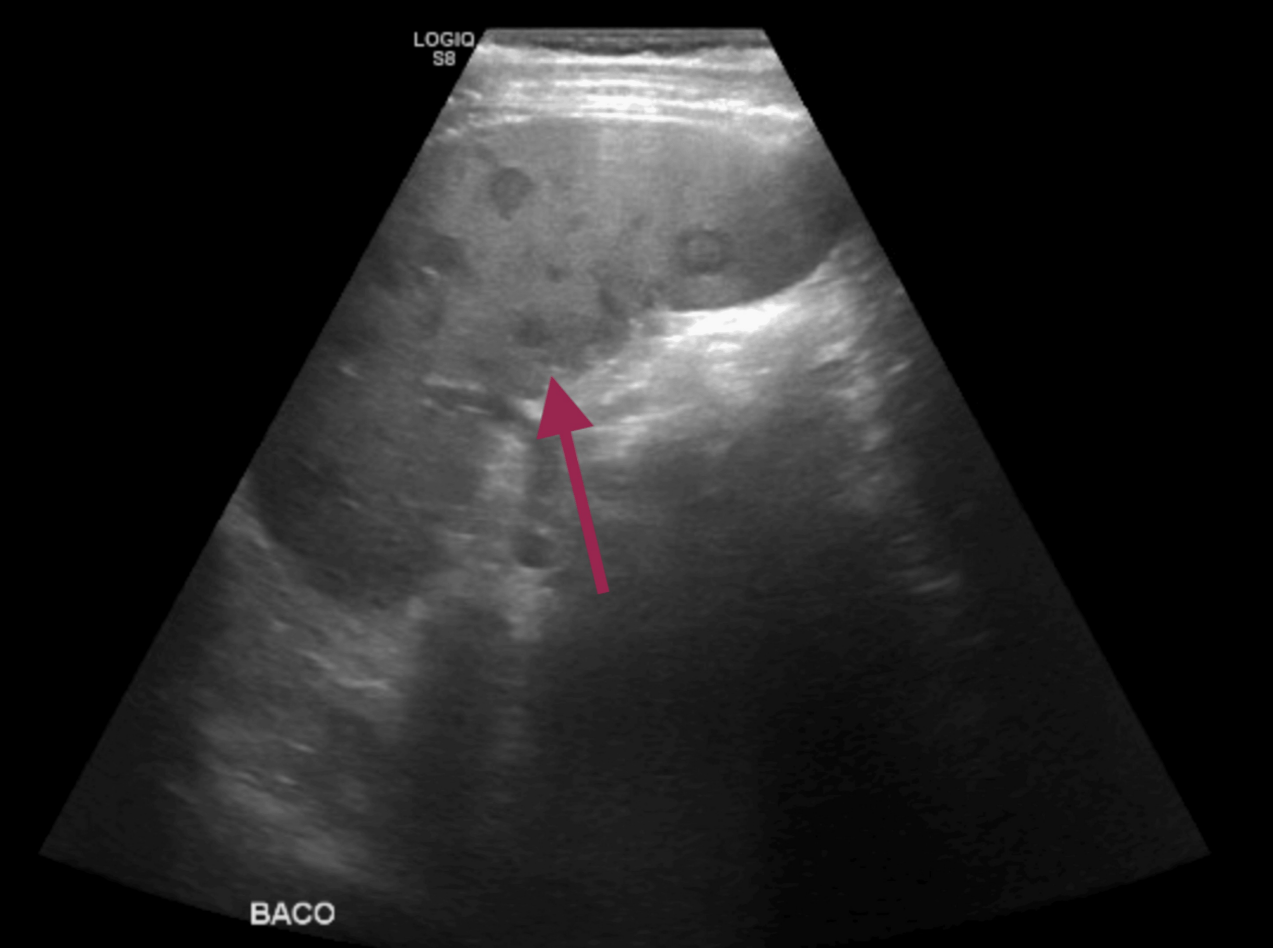
Abdominal ultrasound showing multiple splenic nodules suggestive of infeccious foci Abdominal ultrasound showing splenic nodules that were first thought to be infection but later found to be metastases, after biopsy of one of the hepatic lesions.

**Figure 3:**
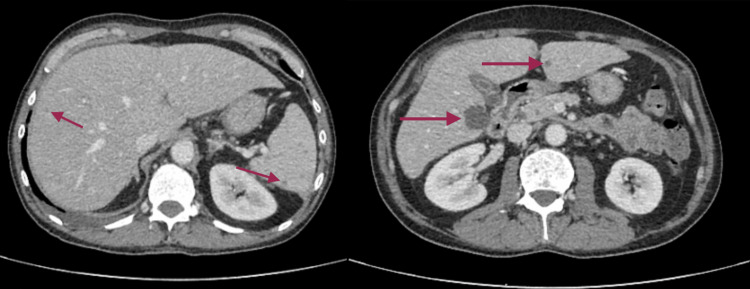
Abdominal CT-scan showing multiple hepatic and splenic nodules suggestive of infeccious foci Abdominal ultrasound showing splenic nodules that were first thought to be infection but later found to be metastases, after biopsy of one of the hepatic lesions.

Due to a progressive increase in leucocyte count and cholestatic parameters, an abdominal ultrasound was requested, revealing multiple hypoechogenic hepatic (Figure 1) and splenic nodules (Figure 2), suggesting infectious foci. A CT scan (Figure 3) was requested for better characterization of the nodules, with a radiology report that suggested infectious semiology, presenting spontaneous hypodensity and hypocaptation on the vascular phases.

As fever and leukocytosis prevailed, despite empiric antibiotics, and considering the possibility of infectious endocarditis given the multiple hepatic and splenic foci, a transthoracic echocardiogram was requested. It was inconclusive, but considering the high possibility of this diagnosis, the patient was initiated on empiric antibiotherapy with ampicillin, ceftriaxone, and gentamicin, and a transesophageal echocardiogram was then requested. This last exam revealed an 18 mm long echogenic mass adherent to the wall of the right ventricular chamber, highly suggestive of being an endocarditis vegetation despite the atypical location.

Blood cultures were once again repeated, and further investigation of blood (Table 2) for bacterial and fungal DNA by polymerase chain reaction (PCR) was requested, all with negative results. In addition to the PCR testing of bacterial and fungal DNA, serologies for the main microorganisms that can cause blood-culture-negative endocarditis, namely *Coxiella burnetii*, *Bartonella* spp, and *Mycoplasma pneumonia* (Table 2), were also requested and were negative. Additionally, due to inflammatory alterations surrounding the lung tumor also revealed in the CT scan, a bronchial fibroscopy was requested for the collection of bronchoalveolar fluid. The microbiologic results (Table 2) were negative for bacteria and fungi, and the nucleic acid amplification test (NAAT) for *Mycobacterium tuberculosis* was also negative. Cultural exams for mycobacteria were collected as well. 

In the presence of five sequential negative blood cultures, and after consulting with Infectious Diseases, Cardiology, and Radiology, it was decided to get a biopsy of the liver abscesses done in order to try to identify the infectious agent. Contrary to expectations, given the different radiology reports suspecting abscesses, the biopsy was positive for a metastasis of pulmonary origin (with immunohistochemistry showing positivity for CK7, TTF-1, and Napsin A, and negativity for CK20), and the fragments sent for culture in the microbiology laboratory were negative.

Once an infectious agent was excluded and the progression of the neoplastic disease documented, the diagnosis of ME was assumed [6]. The patient was discharged on oral anticoagulants, and follow-up with Oncology was scheduled. Unfortunately, the patient was admitted to hospice a month later, due to neoplastic disease progression and a steep fall in palliative performance score, and died two weeks afterwards.

## Discussion

ME, or nonbacterial thrombotic endocarditis (NBTE), is a rare condition most commonly observed in patients with advanced malignancies or hypercoagulable states. While systemic embolic events, such as ischemic strokes, renal or splenic infarctions, are typical, our patient presented without any clinical evidence of embolization, making this case unusual.

The patient’s presentation was atypical for ME, with fever and laboratory findings of neutrophilia and elevated CRP features more suggestive of infective endocarditis. This overlap underscores the diagnostic challenge of ME, a rare but potentially serious condition. As a diagnosis of exclusion, ME necessitates a comprehensive workup to rule out infective endocarditis and other etiologies. The ultrasound and CT findings suggesting liver and splenic infectious foci initially supported an infectious etiology, making the final diagnosis even more challenging.

Autopsy and echocardiographic studies estimate the incidence of ME at 0.3-9.3% of all endocarditis cases, with higher prevalence in oncology patients, reaching up to 10-15% in some series. Echocardiography, particularly transesophageal echocardiography, remains the mainstay imaging modality for detecting valvular vegetations, and the diagnosis can be assumed once infective endocarditis is excluded in patients with predisposing risk factors such as malignancy [7,8]. Blood cultures and molecular testing help differentiate ME from infective endocarditis.

Management primarily involves systemic anticoagulation to reduce thromboembolic risk, with surgical intervention reserved for patients with significant valvular dysfunction or recurrent embolization despite adequate anticoagulation [9]. The goal of treatment is to prevent embolic events, which are the most common and potentially serious complications of ME, particularly strokes [7]. In our patient, no physical signs of embolic events, such as Janeway lesions, were observed. Moreover, the lesions in the liver and spleen initially thought to represent embolic phenomena were ultimately found to be metastases, further emphasizing the diagnostic challenge in distinguishing true embolic events from mimicking lesions.

Despite the absence of embolic events, anticoagulation was initiated. The identification of metastases marked disease progression, after which the patient experienced progressive weight loss and clinical decline. Nevertheless, he was able to spend a full month with his family before transitioning to hospice care.

This case highlights the importance of considering ME even in the absence of embolic events and underscores the need for a thorough and systematic evaluation in cancer patients with unexplained valvular vegetations. Non-embolic cases, like ours, may avoid acute thromboembolic complications, but overall prognosis remains poor and is largely dictated by the underlying malignancy [10].

## Conclusions

We presented the case of a patient with lung cancer, who was diagnosed with marantic endocarditis (ME) after an extensive workup, including blood cultures, serologies, and PCR for bacterial and fungal DNA, which excluded infective endocarditis. The absence of embolic events made the diagnosis particularly challenging, as the clinical picture was confounded by fever, neutrophilia, and elevated inflammatory markers more suggestive of infective endocarditis, and by radiologic findings that initially mimicked embolic foci but were ultimately metastatic lesions.

The patient was treated accordingly with anticoagulation, and no embolic events were reported until his death, which occurred six weeks post diagnosis due to progression of the underlying malignancy. This case underscores the importance of maintaining a high index of suspicion for ME even in the absence of embolic events, particularly in patients with advanced cancer and unexplained valvular vegetations. It also highlights the crucial role of echocardiography, thorough microbiological workup, and systematic exclusion of infective etiologies in reaching the correct diagnosis. This case adds to the growing body of literature demonstrating the heterogeneity of ME presentations and emphasizes that while anticoagulation remains the cornerstone of therapy, the overall prognosis is closely tied to the underlying malignancy rather than to ME itself.
